# Wnt activated β-catenin and YAP proteins enhance the expression of non-coding RNA component of RNase MRP in colon cancer cells

**DOI:** 10.18632/oncotarget.5778

**Published:** 2015-09-22

**Authors:** Jinjoo Park, Sunjoo Jeong

**Affiliations:** ^1^ National Research Lab for RNA Cell Biology, Department of Molecular Biology, Dankook University, Yongin, Gyeonggi-do, Republic of Korea

**Keywords:** RMRP, Wnt signaling, Hippo signaling, YAP, β-catenin

## Abstract

*RMRP*, the RNA component of mitochondrial RNA processing endoribonuclease, is a non-coding RNA (ncRNA) part of the RNase MRP complex functioning in mitochondrial and ribosomal RNA processing. Even though various mutations in the *RMRP* gene are linked to developmental defects and pathogenesis, its relevance to cancer etiology has not been well established. Here we examined the expression of *RMRP* and found a significant increase in colorectal and breast cancer patient tissues. So we tested whether the oncogenic signaling pathways, Wnt/β-catenin and Hippo/YAP pathways, are relevant to the enhanced expression of *RMRP* in cancer cells because of the predicted β-catenin/TCF and YAP/TBX5 elements in the upstream regions of the *RMRP* gene. As expected, Wnt signal activation significantly induced the *RMRP* transcription thru β-catenin and YAP transcription factors. More importantly, YAP protein was critical for *RMRP* transcription by association to the proximal site near the transcription start site of the *RMRP* gene, a Pol III promoter, along with β-catenin and TBX5 proteins. We propose that the interplay of Wnt and Hippo signaling pathways could regulate target genes, coding or non-coding, by the β-catenin/YAP/TBX5 transcription complex in cancer cells.

## INTRODUCTION

Recent advances of transcriptome analysis envision multiple functions for various non-coding RNAs (ncRNAs), but their crucial roles in the survival, proliferation and growth of organisms remain elusive [1, 2]. However, one of the ncRNAs, RNA component of mitochondrial RNA processing endoribonuclease (*RMRP*), is well documented to be essential for normal development and growth, as shown by the incapacitating mutations in cartilage-hair hypoplasia [3, 4]. *RMRP* forms the RNase MRP complex required for pre-rRNA processing to 5.8S rRNA [5, 6] and for mitochondrial RNA processing [7]. In addition to these established functions, additional functions of RNase MRP have been recently proposed [8]. Despite such critical cellular functions of RNase MRP, how the expression level of *RMRP* is regulated by cellular components is not understood at the present time.

Wnt signaling is critical for the development and homeostasis in metazoans with β-catenin being a critical transcription activator [9]. In normal differentiated cells, cytoplasmic β-catenin is phosphorylated by Casein Kinase 1α (CK1α) and Glycogen Synthase Kinase 3β (GSK3β); β-catenin is subsequently degraded in the β-catenin destruction complex composed of Axin and APC (Adenoma Polyposis Coli) [10]. However, upon Wnt signal stimulation, β-catenin accumulates and translocates to the nucleus where it along with the TCF family DNA binding proteins transcribes many target genes. It is now clear that Wnt signal activation is important in the tumorigenesis of various human cancers, some with complicated signaling networks [11].

The Hippo signaling pathway is important for cell death induction, cell proliferation suppression and tissue growth control [12]. The Hippo pathway includes two tumor suppressors, MST1/2 and LATS1/2 kinases, which sequentially phosphorylate transcription factors, YAP and TAZ [13]. Phosphorylated YAP and TAZ are sequestered or degraded in the cytoplasm [14, 15]. However, following Hippo inactivation, dephosphorylated YAP/TAZ proteins can be translocated to the nucleus and activate target genes. So Hippo inactivation status is linked to the tumorigenesis mediated by MST1/2 and LATS1/2 inhibition and YAP/TAZ dephosphorylation [13]. Dysregulation of Hippo signaling is associated with various human cancers, including colorectal cancers [16, 17]. Most significantly, YAP activity is increased in many cancer cells, promoting unrestrained YAP activity counteracting tumor suppressor activities [18].

The extensive interrelationship of Wnt and Hippo signaling pathways has emerged as an important tumorigenic signaling network in some cancer cells [19-21]. In normal cells, YAP and TAZ regulate Wnt signal activation by binding to Disheveled or by associating with the β-catenin destruction complex [22-24]. On the other hand, Wnt signaling inactivates the Hippo signaling pathway by multiple regulatory modules in cancer cells. For example, β-catenin activates transcription of the YAP gene or forms a transcription complex with YAP and TBX5 in the nucleus [25, 26]. To understand the basis of such complicated Wnt and Hippo cancer signaling networks, common and distinct target genes of β-catenin and/or YAP should be identified.

Here we have identified *RMRP* as a common target gene of the Wnt and Hippo signaling pathways. Even though the *RMRP* gene harbors the RNA polymerase III (Pol III) promoter [27], here we demonstrate that *RMRP* transcription is elevated in cancer cells by β-catenin and YAP activation. Especially, the YAP protein is a key transcription factor in this process by associating to the *RMRP* promoter very close to the transcription start site, TATA box. In addition, β-catenin also forms the transcription complex along with YAP and TBX5 at the same site. So it is possible that the interplay of Wnt and Hippo pathways could enhance the tumorigenic potential, probably by *RMRP*-mediated proliferative functions. Moreover, common target genes activated by YAP and β-catenin, such as *RMRP* as shown here, could provide novel oncotargets for cancer therapeutics.

## RESULTS

### RMRP expression level is higher in malignant cells and cancer patient tissues

**Figure 1:**
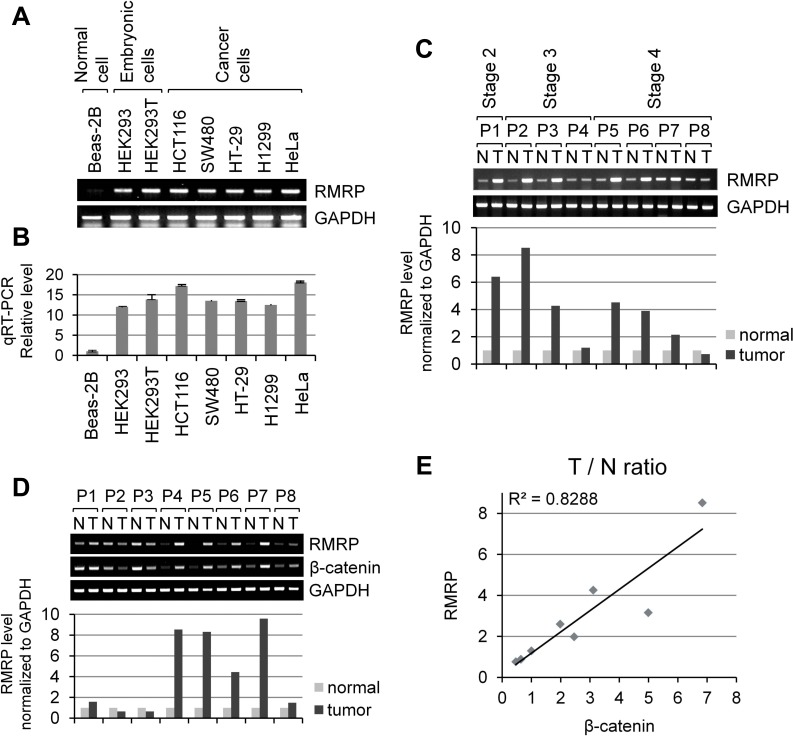
Elevated expression of *RMRP* level in tumor tissues and cancer cells **A.** RT-PCR analysis of *RMRP* levels in various cell lines: Beas-2B (non-malignant lung epithelial cell); HEK293 (embryonic kidney cell), HEK293T (HEK293 cells containing SV40 viral DNA); SW480 and HT-29 (colorectal adenocarcinoma cells); HCT116 (colorectal carcinoma cell); H1299 (lung epithelial, non-small cell carcinoma cells), and HeLa (cervical carcinoma cells). **B.** Relative *RMRP* level in various cell lines detected by qRT-PCR analysis (*n* = 3). **C.** RT-PCR analysis of *RMRP* expression levels in colorectal tumor patient tissues. A bar graph shows a relative level in tumor tissues versus normal tissues. P: patient, N: normal tissue, T: tumor tissue. **D.** RT-PCR analysis of *RMRP* expression levels in breast tumor patient tissues. A bar graph represents a relative RNA levels in tumor tissues versus normal tissues. P: patient, N: normal tissue, T: tumor tissue. **E.** Correlation between *RMRP* and β-catenin mRNA levels from normal and tumor tissues in breast cancer patients. T/N ratio: RNA level in tumor tissues/RNA level in normal tissues.

Since the functions of RNase MRP should be related to the rapid proliferation of cancer cells, we first analyzed the *RMRP* expression level in patient tissues and cancer cells by RT-PCR as well as by real-time qRT-PCR. *RMRP* level was low in nonmalignant lung epithelial Beas-2B cells immortalized by adeno12/SV40 virus. In comparison, higher levels of *RMRP* were detected in embryonic kidney epithelial HEK293 cells, transformed HEK293T cells, and many malignant cancer cell lines, such as colorectal cancer HCT116, SW480 and HT-29, lung cancer H1299 and cervical cancer HeLa (Figure 1A and 1B). Moreover, comparison of patient tissues clearly demonstrated the enhanced expression of *RMRP* in many tumor tissues of colorectal cancer patients (Figure 1C) and in breast tumor patient tissues (Figure 1D), but not in liver tumor patient tissues (Figure S1). We also measured the expression of *CTNNB1* (*β-catenin*) mRNA, which is frequently overexpressed in various cancer patient tissues. Interestingly, enhanced level of *RMRP* was correlated with that of *β-catenin* mRNA in breast cancer tumor tissues (Figure 1E).

### Wnt/β-catenin increases RMRP expression level

**Figure 2:**
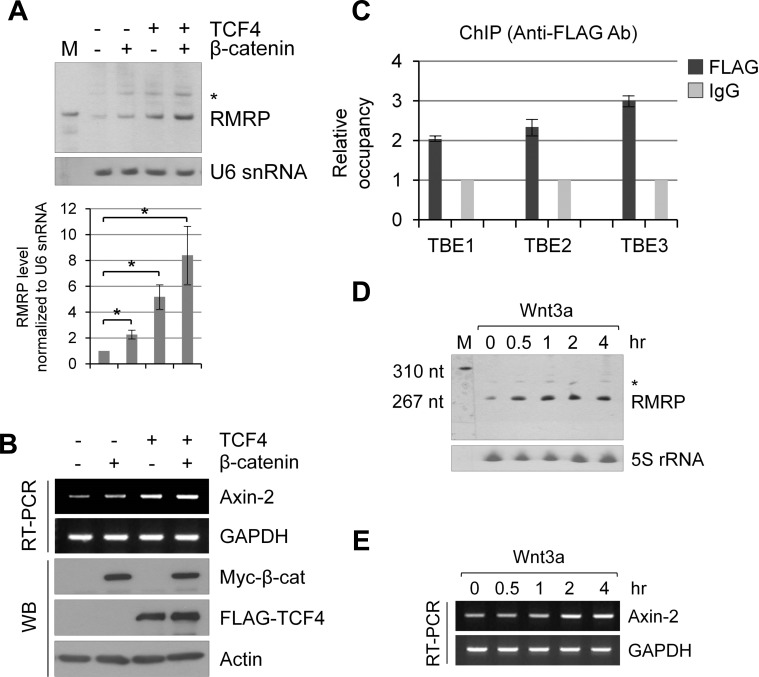
Wnt/β-catenin/TCF4 induced *RMRP* expression **A.** Northern blot analysis of *RMRP* level in HEK293 cells following myc-β-catenin and/or FLAG-TCF4 overexpression. * represents aberrant *RMRP* bands (about 50 bp longer than normal size). A bar graph shows *RMRP* level from three independent experiments. Mean and standard deviation were presented (*p < 0.01). **B.** RT-PCR analysis of Axin-2 mRNA expression levels from the samples of **A.**. **C.** Chromatin immunoprecipitation (ChIP) analysis of *RMRP* promoter using anti-FLAG antibodies following transfection of FLAG-β-catenin in HEK293 cells. Relative occupancy of FLAG-β-catenin in comparison to IgG for three putative TBEs (TCF Binding Elements) upstream of *RMRP* gene are shown. A bar graph shows qPCR analysis of ChIP analysis (*n* = 3). **D.** Northern blot analysis of *RMRP* level in HEK293 cells following Wnt3a treatment for various times (0, 0.5, 1, 2 and 4 hrs). 5S rRNA was used as a control. **E.** Expression of *Axin-2* mRNA as measured by RT-PCR experiments from the samples of **D.**.

The above data suggested a significant correlation of *β-catenin* mRNA with *RMRP* level in cancer patient tissues. Since *β-catenin* mRNA encodes the transcription factor and regulates target genes along with the site-specific DNA binding TCF family transcription factors, we analyzed the upstream region of the RMRP gene. Three putative TCF Binding Elements (TBEs), (A/T)(A/T)CAA(A/T)G, were noticed between 3-4 kb upstream to the transcription start site of the *RMRP* gene (indicated as TBE1, TBE2 and TBE3 in Figure S2A). Northern blot analysis was performed to test whether the overexpression of β-catenin and TCF4 could enhance the expression of *RMRP* (Figure 2A). *RMRP* level was increased by the transfection of β-catenin or TCF4 cDNA, and further enhanced by the co-transfection of both. In contrast, *U6* snRNA expression was not changed in the same samples. To confirm the effect of β-catenin and TCF4 overexpression, transcription from the prototypical target gene, *Axin-2* mRNA, was also analyzed (Figure 2B). To investigate how β-catenin/TCF4 regulates *RMRP* expression, the chromatin immunoprecipitation (ChIP) assay was performed for three TBEs in the upstream of the *RMRP* gene. ChIP experiments clearly showed the FLAG-β-catenin recruitment to TBEs in HEK293 cells (Figure 2C). Since β-catenin/TCF is the key transcriptional complex downstream of Wnt signaling, HEK293 cells were treated with Wnt3a and the expression of *RMRP* was measured by real-time qRT-PCR (Figure 2D, Figure S2B). Rapid induction of *RMRP* level was shown starting at 30 min after Wnt3a treatment, which is faster than the induction of the *Axin-2* mRNA level (Figure 2E). Similarly, lithium chloride treatment, which mimics Wnt signaling by GSK-3 β inhibition, also induced *RMRP* expression (Figure S2C, S2D and S2E).

### Wnt and Hippo activated YAP protein increases RMRP expression level

**Figure 3:**
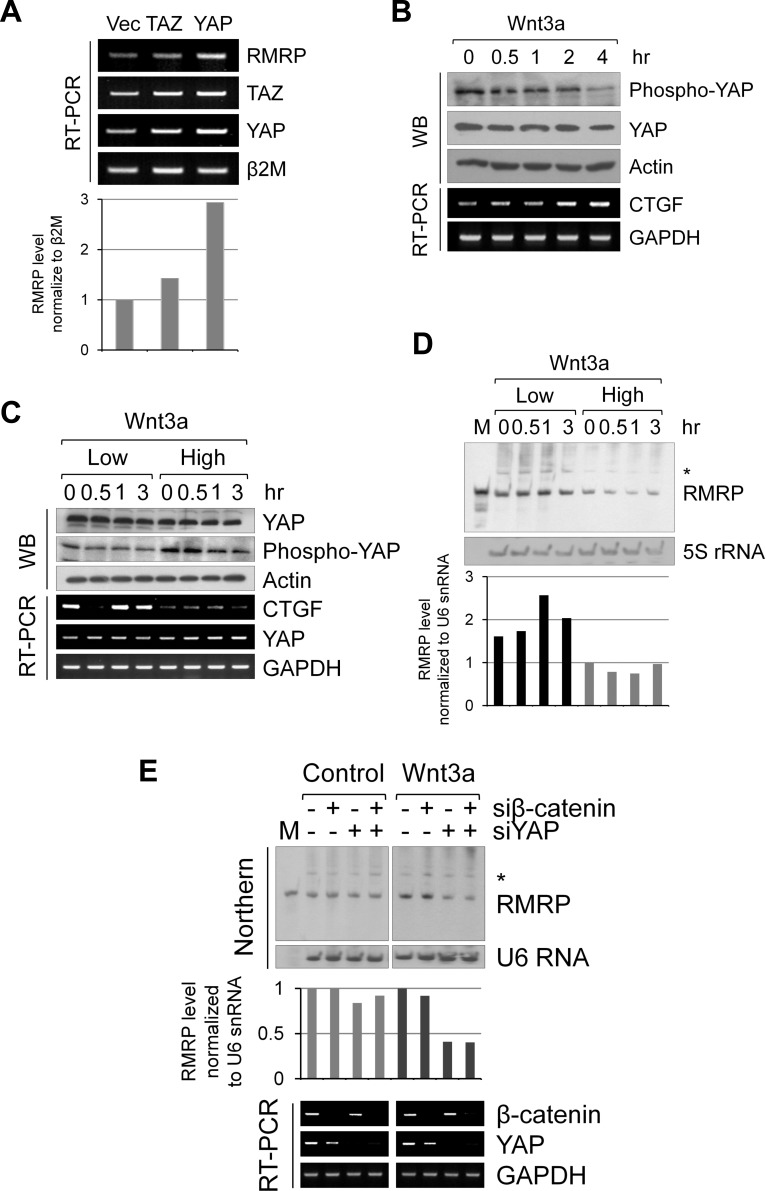
Augmented expression of *RMRP* by Hippo/YAP pathway **A.** RT-PCR analysis of *RMRP* expression levels in Beas-2B cells following transfection of FLAG-TAZ and FLAG-YAP. A bar graph shows densitometer scanning of above gel. **B.** Western blot analysis of YAP protein level and phosphorylation status in HEK293 cells following Wnt3a treatment for various times (0, 0.5, 1, 2 and 4 hrs). RT-PCR analysis indicates YAP target gene *CTGF* mRNA expression levels. **C.** Western blot analysis of YAP protein level and phosphorylation status following Wnt3a treatment (0, 0.5, 1 and 3 hrs) of HEK293 at low and high cell density. RT-PCR analysis of YAP target gene *CTGF* mRNA is also shown. **D.** Northern blot analysis of *RMRP* expression levels from the samples of **C.**. 5S rRNA was also detected as a control. Densitometer scanning of above gel is also shown. **E.** Northern blot analysis of *RMRP* level following Wnt3a or control medium (1hr) treatment to the HEK293 cells silencing with si-β-catenin and si-YAP. A bar graph represents densitometer scanning of above gel. RT-PCR analysis indicates silencing efficiency of the samples.

Recent reports suggest an intricate interplay between Wnt/β-catenin signaling and the Hippo/YAP/TAZ pathway in cancer cells [24, 25, 28]. Since the above data suggested a significant role for Wnt signaling in *RMRP* expression, we tested whether the Wnt ligand regulation of the Hippo pathway components, TAZ or YAP, had any effects in the enhanced expression of *RMRP* [28]. Interestingly, TAZ overexpression never changed *RMRP* expression level; however, YAP protein strongly enhanced *RMRP* expression (Figure 3A). Notably, Wnt signaling consistently activated YAP as shown by the dephoshphrylation of YAP protein and an induction of the YAP target gene, *CTGF* mRNA (Figure 3B). Strikingly, the Wnt signal induction of YAP protein activation was more dramatic at the lower cell density (Figure 3C). Consistent with a cell density-regulated Hippo/YAP signaling, *RMRP* level was more enhanced at low cell density where prominent YAP activation occurred (Figure 3D). To test whether Wnt-activated β-catenin and YAP proteins were essential for *RMRP* expression, silencing with siRNAs for β-catenin and/or YAP was carried out in HEK293 cells. At the basal level, without Wnt3a treatment, the *RMRP* level was not changed either by β-catenin or by YAP silencing. However, following Wnt3a treatment, *RMRP* expression level was decreased by YAP silencing (Figure 3E), suggesting that YAP protein plays an important role in enhancing Wnt-induced *RMRP* expression. Therefore, we conclude that the YAP protein activation induced by Wnt signaling or by low cell density could regulate *RMRP* expression in normal cells.

### YAP protein strongly associates to the proximal site of the RMRP promoter

**Figure 4:**
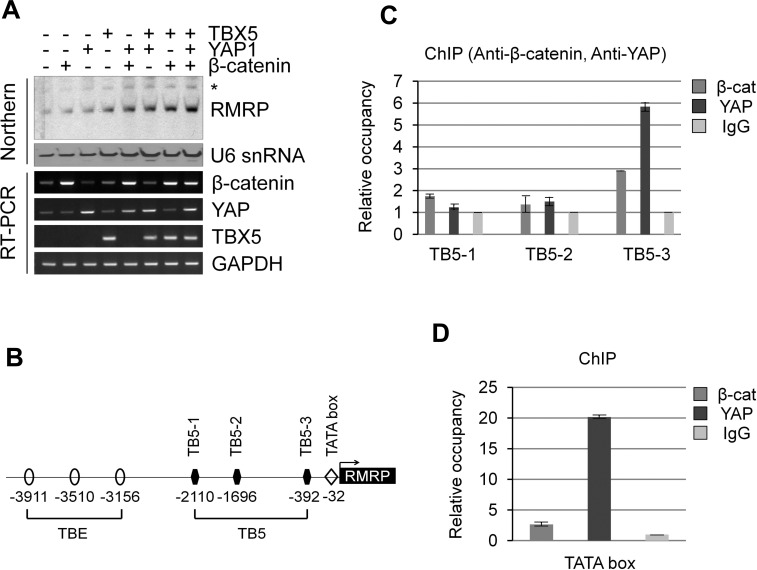
Chromatin association of β-catenin/YAP/TBX5 transcription complex to *RMRP* promoter **A.** Northern blot analysis of *RMRP* level following transfection of FLAG-β-catenin, FLAG-YAP and FLAG-TBX5 in HEK293 cells. A bar graph represents densitometer scanning of above gel. RT-PCR analysis indicates DNA transfection efficiency. **B.** Diagram indicating three TBE (TCF Binding Element), three TB5 (TBX5 Binding site) and TATA box in the *RMRP* promoter. An arrow indicates transcription start site. **C.** ChIP analysis using anti-β-catenin and anti-YAP antibodies in colon cancer SW480 cells. IgG was used as a negative control. qPCR analysis is shown by a graph indicating β-catenin or YAP binding to three TBX5 binding sites (TB5-1, -2 and -3). **D.** ChIP and qPCR analyses as above to TATA box of *RMRP* promoter.

Wnt/β-catenin signaling and the Hippo pathway might be interconnected by sharing a transcriptional complex, as proposed for β-catenin/YAP/TBX5 to be important for tumor cell survival and tumorigenesis in β-catenin-driven cancer [26]. Interestingly, overexpression of TBX5 alone increased *RMRP* expression, but more dramatic enhancement of the *RMRP* level was shown by the combination of β-catenin and YAP (Figure 4A). The RMRP promoter was known to be the type 3 RNA polymerase III promoter [27]. We noticed three putative TBX5 binding sites (named as TB5-1, -2 and -3) proximal to the transcription start site of the *RMRP* gene (Figure 4B). Consequently, ChIP analysis was performed to test the association of the β-catenin and YAP protein to the predicted TBX5 sites in colon cancer SW480 cells in which Wnt signaling was activated. Strikingly, β-catenin and YAP associated with the most proximal site, TB5-3 (located at −392), of the transcription start site with more strong enrichment of YAP protein (Figure 4C and Figure S3A). Recruitments of β-catenin and YAP to TB5-3 in Wnt activated cells were confirmed by ChIP with anti-FLAG antibody following FLAG- β-catenin or FLAG-YAP expression in HEK293 cells (Figure S3B) [30, 31]. Interestingly, YAP protein association was induced by Wnt activation, but β-catenin was not. Most strikingly, prominent enrichment of YAP protein was at the TATA box (−32), very close to the transcription start site (Figure 4D). Thus, we conclude that the YAP/β-catenin/TBX5 transcription complex associates very closely to the Pol III type RMRP promoter.

### Intracellular localization of RMRP is regulated by Wnt activation

**Figure 5:**
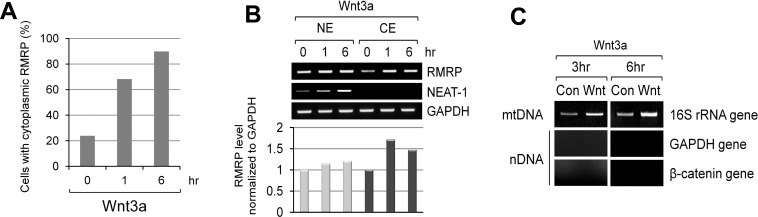
Increase of cytoplasmic *RMRP* level following Wnt3a treatment **A.** Quantitation of Fluorescence In Situ Hybridization (FISH) data as shown in Figure S4B. HeLa cells were stained with *RMRP* probe and the percentage of cells with cytoplasmic *RMRP* was counted from the stained cells (*n* = 65). **B.** Cellular fractionation analysis of *RMRP*. Nuclear and cytoplasmic levels of *RMRP* from the fractionated HeLa cells following Wnt3a treatment. A graph represents densitometer scanning of above gel. **C.** PCR analysis of mitochondrial DNA (mtDNA). Mitochondrial fractions were prepared and mtDNA was purified. Mitochondrial 16S rRNA gene was detected to measure the amount of mtDNA. Nuclear DNA (nDNA) contamination was also monitored by *GAPDH* and β-catenin genes. Wnt3a treatment for 3 hrs was executed in HeLa cells.

Since Wnt signaling enhanced the expression level of *RMRP*, we next tested whether the intracellular localization of *RMRP* was also regulated by Wnt3a treatment. As shown by in situ hybridization, RMRP was highly localized to the nucleolus, and found some in the nucleoplasm (Figure S4A and S4B). More significantly, a percentage of the cells containing cytoplasmic RMRP was clearly increased following Wnt3a treatment (Figure 5A). Cellular fractionation analysis also confirmed the cytoplasmic localization of RMRP by Wnt activation (Figure 5B). Since *RMRP* forms the RNase MRP complex in the cytoplasm for mitochondrial RNA processing activity [32], we tested whether Wnt3a treatment could also enhance such RNase MRP functions. Strikingly, mitochondrial genome copies were increased after Wnt3a treatment as shown by mitochondrial fractionation and mitochondrial DNA (mtDNA) purification (Figure 5C). 16S mitochondrial rRNA gene was also used as a marker for mtDNA (Figure S4C). Thus, it is likely that the Wnt induction of cytoplasmic *RMRP* somehow promoted mitochondrial DNA amplification, possibly related to RNase MRP function.

## DISCUSSION

**Figure 6:**
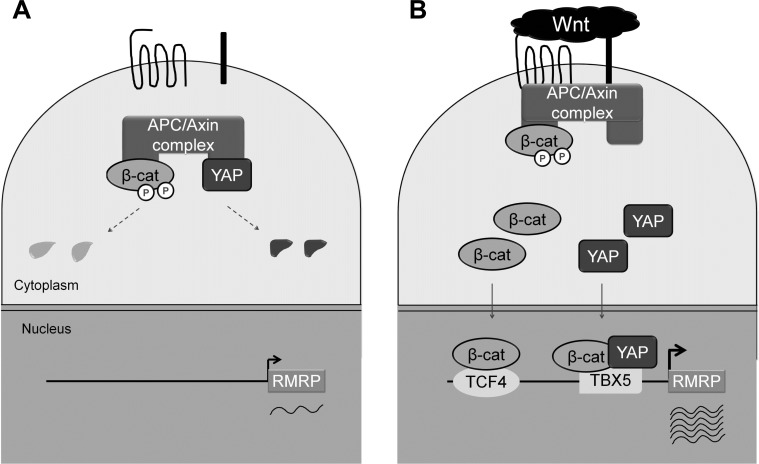
Model for Wnt enhanced *RMRP* expression **A.** Without Wnt activation, constitutive level of *RMRP* is expressed. β-catenin and YAP proteins are low in Wnt OFF state due to the degradation in the cytoplasm. **B.** Upon Wnt activation, β-catenin and YAP protein levels are increased and translocated to the nucleus. Association of β-catenin and YAP to the *RMRP* promoter in conjunction with DNA binding proteins, TCF and TBX5, lead to the enhanced expression of *RMRP*.

Here we have shown that the *RMRP* level is enhanced in cancer cell lines and tumor tissues by Wnt-activated β-catenin and YAP proteins. Having a couple of putative TCF and TBX5 binding elements in the upstream regions of the *RMRP* gene, β-catenin and YAP proteins play critical roles as shown in the Model (Figure 6). In normal cells, the basal transcription of the *RMRP* gene occurs when β-catenin and YAP proteins are degraded or sequestered in the cytoplasm (Figure 6A). However, in cancer cells Wnt stimulation increases the protein level of β-catenin and YAP in the nucleus and recruits them to the promoter regions of the *RMRP* gene via TCF4 and TBX5 DNA binding proteins, which leads to elevated transcription of *RMRP* (Figure 6B). β-catenin generally activates target genes by forming a transcription complex with TCF/LEF DNA binding proteins, as for the distal region of the *RMRP* gene (Figure 2). However, in the proximal region of the *RMRP* gene, β-catenin interacts also with YAP and TBX5 proteins, forming a triple transcription complex on the promoter [26]. Further experiments are needed to more clearly demonstrate the role of Wnt activated YAP for *RMRP* expression.

Even though the *RMRP* gene is known to be transcribed by Pol III [27], more complex regulation is likely to be necessary for RMRP transcription in cancer cells [26]. So far, β-catenin and YAP are known to be the transcriptional co-activators of Pol II transcription. Intriguingly, the ChIP experiment in this study clearly demonstrated the dramatic enrichment of YAP and β-catenin at TBX5 binding sites, and more strong occupancy of YAP to the TATA box near the Pol III transcription start site (Figure 4). Pol III transcription is generally active in transformed cells by overexpressing the BDP1 subunit of TF III B and all the subunits of TF III C2 [33]. Also, the tumor suppressor protein retinoblastoma (RB) and p53 inhibits Pol III transcription by binding to TFIIIB [34]. On the other hand, the oncogene products, c-myc and Erk, bind TF III B, but in this case activate Pol III transcription [34]. So it would be important to elucidate how YAP and β-catenin enhance the Pol III transcription. Since a DNA binding protein like TBX5 is likely to be associated with the TATA box, it is tempting to speculate that YAP activated *RMRP* enhancement is mediated by direct activation of TFIIIB. In addition, it will be interesting to test whether Pol II is involved in the transcription of the *RMRP* gene depending upon cellular context, such as in cancer cells. Additionally, the identification of more common target genes, coding or non-coding, of the Wnt and Hippo pathways would be expected to generate more critical oncotargets.

*RMRP* forms a stable ribonucleoprotein (RNP) with 10 different proteins and plays essential roles during development and growth [35]. Considering dual roles of RNase MRP in the nucleolus and in the mitochondria, developmentally regulated expression and localization of *RMRP* seem to be relevant to the regulation of two distinct functions [36, 37]. Conditional silencing of *RMRP* or heterozygous null of *RMRP* in mouse clearly demonstrates a developmental lethal phenotype [3]. In human, many mutations of the *RMRP* gene have been reported, some of which are in the promoter region of the *RMRP* gene [38]. Since most these *RMRP* mutations cause the reduction in *RMRP* level and associate with cartilage-hair hypoplasia, reduced functions of the RNase MRP complex contribute to the pathogenesis [4]. Even though the amount of *RMRP* is increased upon Wnt activation, it is not clear at this moment whether additional RNase MRP is assembled upon Wnt stimulation. However, the increase of mitochondrial and ribosomal processing activities indirectly points to the fact that functional RMRP is likely to be generated by Wnt activation. Recent studies propose that *RMRP* can play a critical role as the alternative RNP complex with hTERT (Telomerase Reverse Transcriptase) acting as a RNA-dependent RNA polymerase [8, 39]. It will also be interesting to test whether such newly emerged forms of alternative RNP complex with *RMRP* can be induced by Wnt stimulation [8].

Diverse sets of non-coding RNAs, either short or long, have been discovered recently [2]. Some of lncRNAs, such as HOTAIR and MALAT-1, are proposed to promote tumor cell metastasis [40, 41], but further functional tests are required. In contrast, functions of *RMRP* contribute to proper growth in humans so that the tight regulation of the expression level and localization of *RMRP* might be linked to normal human development [3]. Therefore, we could infer that uncontrolled activation of Wnt signaling in cancer cells leads to unregulated *RMRP* and other ncRNA enhancement via nuclear β-catenin and the YAP protein, all of which could be relevant to tumor cell formation and progression.

## MATERIALS AND METHODS

### Cell culture and patient tissues

Beas-2B lung transformed cells (non-malignant thoracic bronchial epithelial cells immortalized by adeno12/SV40 virus) were cultured in defined keratinocyte serum-free medium (Gibco) at 37°C in 5% CO_2_ incubator. Embryonic kidney epithelial HEK293 and HEK293T (HEK293 cells containing SV40 viral DNA) were cultured in MEM medium (Hyclone). SW480 and HT-29 (colorectal adenocarcinoma cells), HCT116 (colorectal carcinoma cell) and H1299 (lung epithelial, non-small cell carcinoma cell) were cultured in RPMI 1640 medium (Hyclone), HeLa cells (cervical cancer cell) were cultured in DMEM high glucose medium (Hyclone) with 10% FBS and 1x antibiotics (Hyclone) at 37°C in 5% CO_2_ incubator. Matched pairs of primary tumor and adjacent normal tissue samples were obtained from the Liver Cancer Specimen Bank, supported by the National Research Resource Bank Program of the Korea Science and Engineering Foundation in the Ministry of Science and Technology. Consent to use the individual tissue specimens for research purposes was obtained from the patients, and the utilization of the specimens for this research was authorized by the Institutional Review Board of College of Medicine, Yonsei University, Korea (provided from Dr. Hogeun Kim)

### Quantitative RT-PCR

RNA was isolated using Trizol reagent (Molecular Research Center; MRC). For reverse transcription, 2 ug of total RNA was used. StepOne^TM^ real time PCR systems (Life Technologies) was used for real-time PCR. Primers for PCR were listed in Supplementary Table 1.

### Western blot analysis

Cells were lysed in RIPA lysis buffer (50 mM Tris-HCl, pH 8.0, 150 mM NaCl, 1% NP-40, 0.5% sodium deoxycholate, 0.1% SDS). After loading proteins on the 8-12% SDS-PAGE gel, they were transferred to PVDF membrane (BMS). Antibodies used for this research were as listed as below: β-catenin (Epitomics #1247-1 and BD biosciences) used with 1:3000, YAP (Cell Signaling #4912) used with 1:500, phospho-YAP (Ser127) (Cell Signaling #4911) used with 1:1000, Lamin B1 (Abcam ab16048) used with 1:5000, β-actin (Abcam ab6276) used with 1:10000, Myc-tag (Millipore 05-724) used with1:3000, FLAG (Sigma F3165) used with 1:3000.

### Northern blot analysis

DIG Northern Starter Kit (Roche) was used for non-radioactive northern blot analysis. RNA was loaded on the denaturing 7 M urea-6% polyacrylamide gel and transferred to the Nylon membranes (Roche) for 1 hour. After UV cross-linking, the probes were treated on membranes and hybridized overnight. Washing was as follows: 2xSSC and 0.1% SDS buffer at room temperature for 5 min twice under constant agitation, second washing was in 0.1xSSC and 0.1% SDS buffer at 68°C for 15 min three times under constant agitation. With anti-digoxigenin-AP antibody, AP reaction was carried out for more than 30 min. The probes used for this study were *RMRP* (100 ng/ml), *5.8S* ribosomal RNA (5 ng/ml), *U6* small nuclear RNA (100 ng/ml) and *5S* ribosomal RNA (6 ng/ml). List of probes is shown in Supplementary Table 2.

### Immunocytochemistry and fluorescence in situ hybridization

After fixing, cells were permeablized with 0.5% Triton X-100 in 1x PBS buffer for 10 min. Blocking was carried out for 1 hour in 5% normal horse serum in 1x PBS, then primary antibodies were added onto the coverslips for immunocytochemistry. Secondary antibodies were treated onto coverslips for 1 hour. For FISH, *RMRP* probe (100nM, 5′-gcctgcgtaactagagggagc-3′) was added onto the coverslip and the hybridization was carried out overnight. Washing was done in SSC buffer.

### Chromatin immunoprecipitation

After fixing cells with 1% formaldehyde for 15 min, cells were treated by 125 mM glycine for 10 min. Cells were lysed in ChIP RIPA buffer (50 mM Tris-HCl, pH 8.0, 150 mM NaCl, 1 mM EDTA, 1% NP-40, 1% sodium deoxycholate, 0.05% SDS, 1x protease inhibitor cocktail) and sonication was carried out (amplitude 60%, pulse time 20 sec, 6 times). Protein G sepharose beads (GE healthcare) were mixed with cell lysates for pre-clearing and 500 ug proteins were mixed with 2-3 ug antibodies. Washing was carried out 5 times in low salt buffer (0.1% SDS, 1% Triton X-100, 2 mM EDTA, 20 mM Tris-HCl, pH 8.1, 150 mM NaCl), high salt buffer (0.1% SDS, 1% Triton X-100, 2 mM EDTA, 20 mM Tris-HCl, pH 8.1, 500 mM NaCl) once, LiCl wash buffer (250 mM LiCl, 0.5% NP-40, 0.5% sodium deoxycholate, 1 mM EDTA, 10 mM Tris-HCl, pH 8.0) once, TE buffer (10 mM Tris-HCl, pH 8.0, 1 mM EDTA) twice. DNA was eluted in elution buffer (50 mM Tris-HCl, pH 8.0, 10 mM EDTA, 1% SDS) and RNA was removed. To revert the cross-linking, the samples were incubated at 65°C overnight. StepOne^TM^ real time PCR systems (Life Technologies) was used for real-time PCR of precipitated DNA.

### Cellular fractionation

Cells were added in RSB buffer (10 mM Tris-HCl, pH 7.4, 10 mM NaCl, 3 mM MgCl_2_, 1x protease inhibitor cocktail, RNase inhibitor 100 units/ml) and then 0.4 ug/ml digitonin was added to the tubes. After 10 min incubation in ice, the samples were centrifuged at 4,400 rpm for 8 min at 4°C. Cytoplasmic extract was obtained from the supernatant. RIPA lysis buffer (50 mM Tris-HCl, pH 8.0, 150 mM NaCl, 1% NP-40, 0.5% sodium deoxycholate, 0.1% SDS) was added to the pellet and then the samples were incubated in ice for 15 min. They were centrifuged at 13,200 rpm for 15 min at 4°C. Nuclear extract was obtained from the supernatant.

### Mitochondrial DNA purification

Homogenization buffer (0.32 M sucrose, 1 mM EDTA, 10 mM Tris-HCl, pH 7.8) was added to the cells, it was incubated in ice for 10 min. Homogenization was carried out using homogenizer with 5,000 rpm ten times. Cell lysates were centrifuged at 1,000 x g for 10 min at 4°C. The supernatant was centrifuged at same condition, followed by being centrifuged at 13,000xg for 20 min at 4°C. Homogenization buffer was added to the pellet and then it was centrifuged at 13,000 x g for 10 min at 4°C. Lysis buffer (10 mM Tris-HCl, pH 8.0, 150 mM NaCl, 20 mM EDTA, 1% SDS, 0.2 mg/ml proteinase K) was added to the pellet. After incubation at 55°C for 1 hour, phenol extraction was carried out three times. The supernatant was mixed with same volume of isopropanol and 30 ug glycogen, and centrifuged at 13,000xg for 15 min at 4°C. Mitochondrial DNA was obtained from the pellet.

## SUPPLEMENTARY MATERIAL TABLE AND FIGURES


