# Supplementary material for: Wnt activated β-catenin and YAP proteins enhance the expression of non-coding RNA component of RNase MRP in colon cancer cells
Source: Oncotarget. 2015 Sep 22;6(33):34658–68. doi: 10.18632/oncotarget.5778 (PMC4741480; doi:10.18632/oncotarget.5778)
Supplement: Supplementary file 1 [file oncotarget-06-34658-s001.pdf]

Wnt activated β-catenin and YAP proteins enhance the expression of non-coding RNA component of RNase MRP in colon cancer cells

Supplementary Material

Supplementary Table 1. List of RT-PCR primers

Target	Type	Sequence
RMRP	For	5' – CGTGCTGAAGGCCTGTATCC – 3'
	Rev	5' – GCTTCTTGGCGGACTTTGG – 3'
β-catenin	For	5' – CGGGATCCACAAGAAACGGCTTTCA – 3'
	Rev	5' – GAGAATTCCAGGTCAGTATCAAACCA – 3'
YAP	For	5' – AGCAGGATGGTGGGACTCAA – 3'
	Rev	5' – TGAGGGCAGGGTGCTTTGG – 3'
TBX5	For	5' – CCAGCCTAGATTACACATCGTG – 3'
	Rev	5' – TGTGGTTCTGGTAGGAAGTCAC – 3'
TCF4	For	5' – CAAAGACGAGGGCGAACAGG – 3'
	Rev	5' – TGGAGATAGGTTCGGGCGGT – 3'
Axin-2	For	5' – GCTAC AGGAAGACCCGCAG – 3'
	Rev	5' – TAGTGGTGG TGGACATGCTTCG – 3'
CTGF	For	5' – AAGACCTGTGGGATGGGCA – 3'
	Rev	5' – CAAACTTGATAGGCTTGGAGATT – 3'
TAZ	For	5' – CACCCACGATGACCCCAGA – 3'
	Rev	5' – GCCAGGTTAGAAAGGGCTCAC – 3'
c-myc	For	5' – AGGCTCCTGGCAAAAGGTCA – 3'
	Rev	5' – ACATTCTCCTCGGTGTCCGA – 3'
hTERT	For	5' – CGTCCAGTGCCAGGGG – 3'
	Rev	5' – CGTGGGTGAGGTGAGGTGT – 3'
GAPDH	For	5' – TGACATCAAGAAGGTGGTGA – 3'
	Rev	5' – TCCACCACCCTGTTGCTGTA – 3'
β2M	For	5' – CATCCATCCGACATTGAAGTTG – 3'
	Rev	5' – ATGGTTCACACGGCAGGCA – 3'

Supplementary Table 2. List of northern probes

RNA name	Location (nt)	Length (nt)	Sequence
RMRP	5 - 266	262	CGTGCTGAAGGCCTGTATCCTAGGCTACACA<br>CTGAGGACTCTGTTCCTCCCCTTTCCGCCTAG<br>GGGAAAGTCCCCGGACCTCGGGCAGAGAGT<br>GCCACGTGCATACGCACGTAGACATTCCCCG<br>CTTCCCACTCCAAAGTCCGCCAAGAAGCGTAT<br>CCCGCTGAGCGGCGTGGCGCGGGGGCGTCA<br>TCCGTCAGCTCCCTCTAGTTACGCAGGCAGT<br>GCGTGTCCGCGCACCAACCACACGGGGCTCA<br>TTCTCAGCGCGGCT
5.8S rRNA	2 - 156	155	ACTCTTAGCGGTGGATCACTCGGCTCGTGCG<br>TCGATGAAGAACGCAGCTAGCTGCGAGAATTA<br>ATGTGAATTGCAGGACACATTGATCATCGACA<br>CTTCGAACGCACTTGCGGCCCCGGGTTCCTC<br>CCGGGGCTACGCCTGTCTGAGCGTCGCTT
U6 snRNA	1 - 106	106	GTGCTCGCTTCGGCAGCACATATACTAAAATT<br>GGAACGATACAGAGAAGATTAGCATGGCCCC<br>TGCGCAAGGATGACACGCAAATTCGTGAAGC<br>GTTCCATATTTT
5S rRNA	1 - 120	120	GTCTACGGCCATACCACCCTGAACGCGCCCG<br>ATCTCGTCTGATCTCGGAAGCTAAGCAGGGTC<br>GGGCCTGGTTAGTACTTGGATGGGAGACCGC<br>CTGGGAATACCGGGTGCTGTAGGCTT

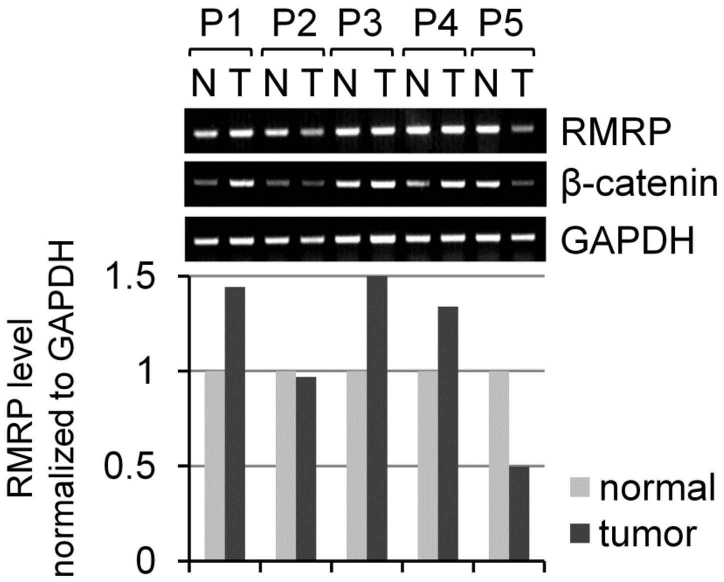

Supplementary Figure 1. *RMRP* expression in liver cancer patient tissues.

RT-PCR analysis of *RMRP* expression levels in liver tumor patient tissues. A bar graph shows the relative level in tumor tissues versus normal tissues. P: patient, N: normal tissue, T: tumor tissue.

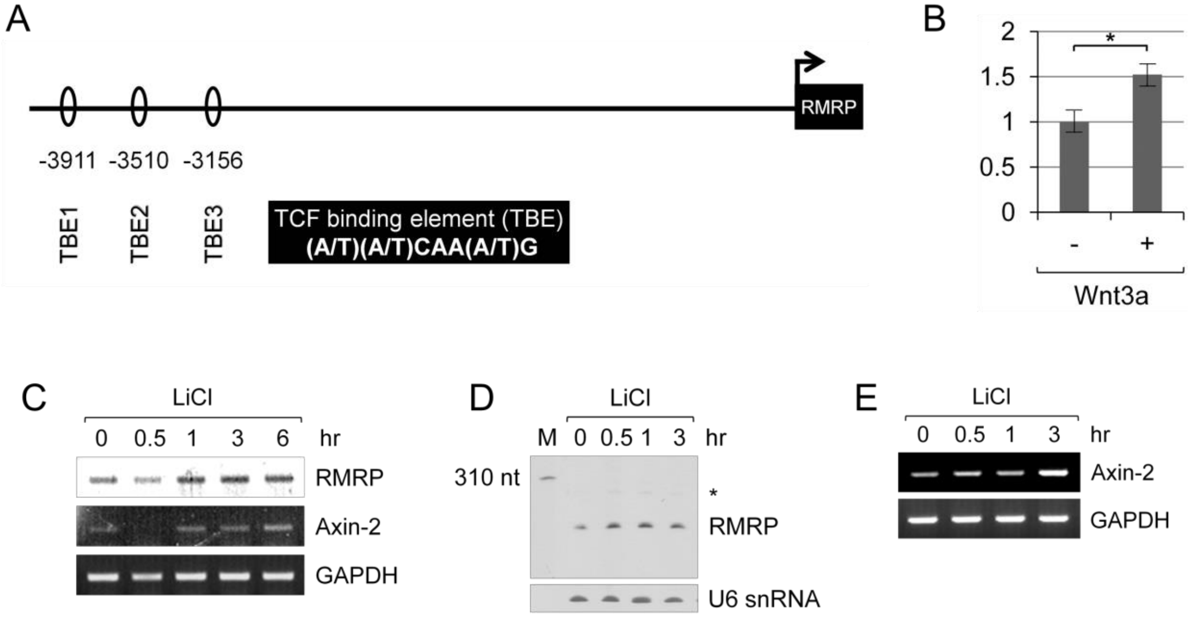

Supplementary Figure 2. Predicted β-catenin/TCF sites in *RMRP* promoter and Wnt or LiCl activated *RMRP* expression level.

(A) Diagram of the *RMRP* promoter with three putative TBEs (TCF Binding Elements) upstream of the *RMRP* promoter. An arrow indicates the transcription start site of the *RMRP* gene. (B) Quantitative real-time PCR analysis of *RMRP* level in HEK293 cells following Wnt3a treatment for 1 hour. A bar graph shows *RMRP* level from three independent experiments. Mean and standard deviation is shown (*$p < 0.01$). (C) RT-PCR analysis of *RMRP* expression following lithium chloride (20 mM) treatment for 0, 0.5, 1, 3 and 6 hrs in Beas-2B cells. (D) Northern blot analysis of *RMRP* expression in HEK293 cells following lithium chloride (20 mM) treatment for 0, 0.5, 1 and 3 hrs. (E) RT-PCR analysis of *Axin-2* mRNA expression from the samples of (D).

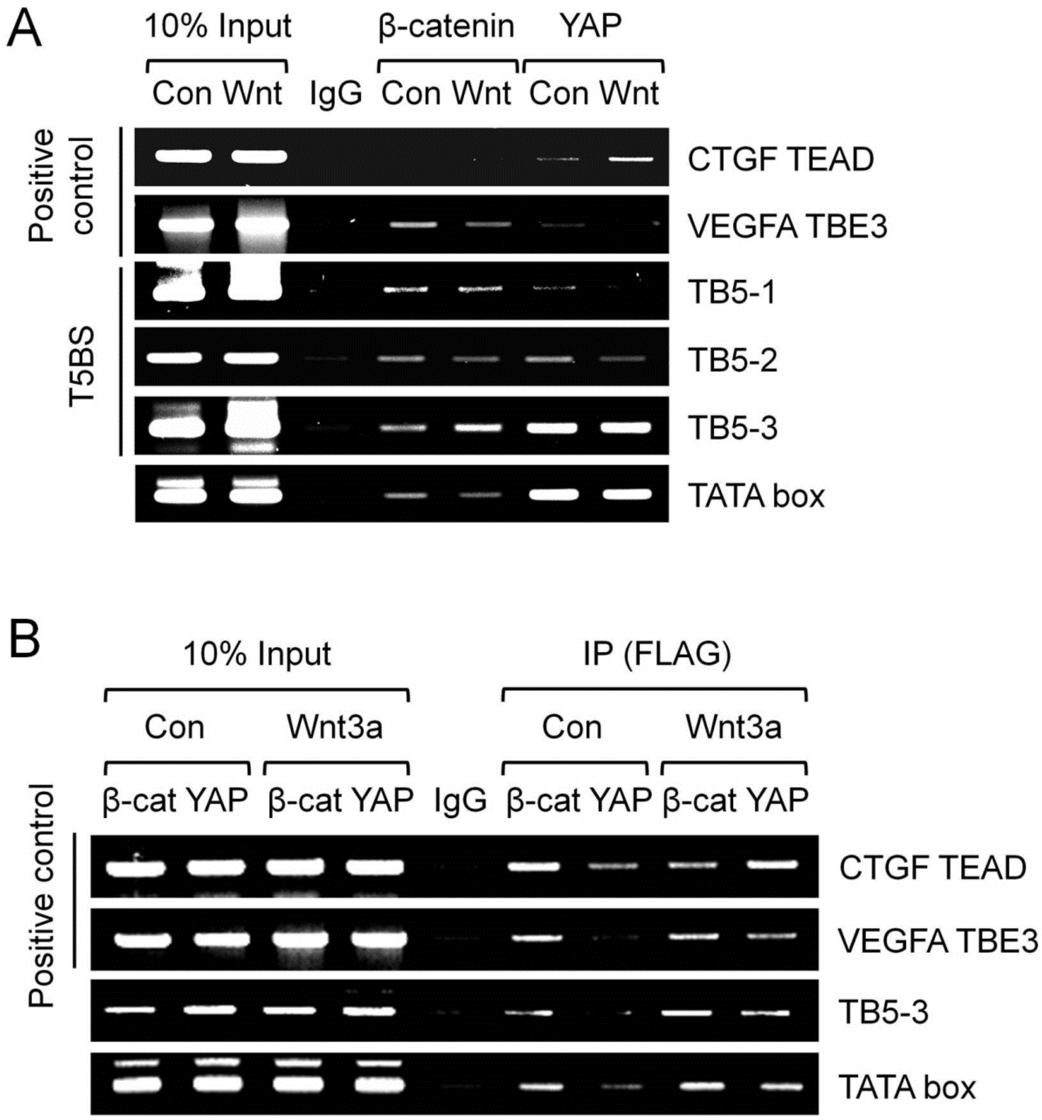

Supplementary Figure 3. β-catenin and YAP associate to TBX5 binding sites and TATA box of *RMRP* promoter.

(A) Chromatin immunoprecipitation (ChIP) analysis using anti-β-catenin and anti-YAP antibodies in SW480 cells. TBX5 binding sites (TB5) and the TATA box of the *RMRP* promoter were tested for the recruitment of β-catenin or YAP. Recruitment of β-catenin or YAP is shown by ChIP on TEAD binding site in the *CTGF* promoter and on TBE3 (TCF Binding Elements 3) in the VEGFA promoter, respectively. (B) ChIP analysis using anti-FLAG antibodies in HEK293 cells following transfection of FLAG-β-catenin and FLAG-YAP in HEK293 cells

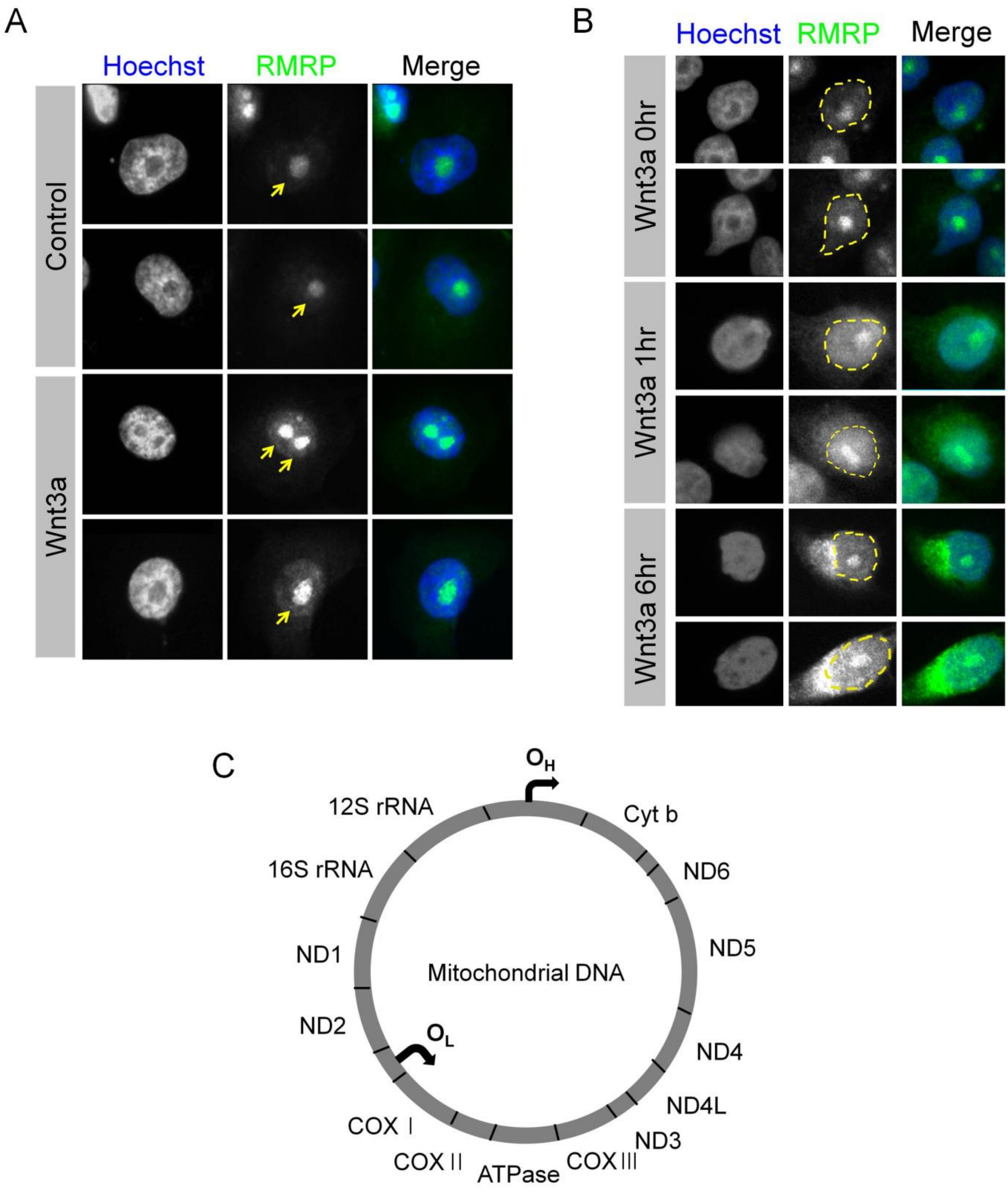

Supplementary Figure 4. Wnt signaling could enhance mitochondrial DNA replication.

(A) Fluorescence in situ hybridization (FISH) analysis of *RMRP* (green) after Wnt3a treatment for 1 hr in H1299 cells. Yellow arrows indicate the nucleolus of cells. (B) FISH analysis of *RMRP* (green) after Wnt3a treatment for 1 or 6 hrs in HeLa cells.

Yellow lines indicate nuclear boundaries. (C) Diagram of mitochondrial genome. PCR primers designed for 16S ribosomal RNA gene were used.
